# Defects in Mitochondrial Dynamics and Metabolomic Signatures of Evolving Energetic Stress in Mouse Models of Familial Alzheimer's Disease

**DOI:** 10.1371/journal.pone.0032737

**Published:** 2012-02-29

**Authors:** Eugenia Trushina, Emirhan Nemutlu, Song Zhang, Trace Christensen, Jon Camp, Janny Mesa, Ammar Siddiqui, Yasushi Tamura, Hiromi Sesaki, Thomas M. Wengenack, Petras P. Dzeja, Joseph F. Poduslo

**Affiliations:** 1 Department of Molecular Pharmacology and Experimental Therapeutics and Neurology, Mayo Clinic, Rochester, Minnesota, United States of America; 2 Cardiovascular Diseases, Mayo Clinic, Rochester, Minnesota, United States of America; 3 Electron Microscopy Core Facility, Mayo Clinic, Rochester, Minnesota, United States of America; 4 Biomedical Imaging Resource, Mayo Clinic, Rochester, Minnesota, United States of America; 5 Department of Cell Biology, Johns Hopkins University School of Medicine, Baltimore, Maryland, United States of America; 6 Departments of Neurology and Biochemistry and Molecular Biology, Mayo Clinic, Rochester, Minnesota, United States of America; The Mental Health Research Institute of Victoria, The University of Melbourne, Australia

## Abstract

**Background:**

The identification of early mechanisms underlying Alzheimer's Disease (AD) and associated biomarkers could advance development of new therapies and improve monitoring and predicting of AD progression. Mitochondrial dysfunction has been suggested to underlie AD pathophysiology, however, no comprehensive study exists that evaluates the effect of different familial AD (FAD) mutations on mitochondrial function, dynamics, and brain energetics.

**Methods and Findings:**

We characterized early mitochondrial dysfunction and metabolomic signatures of energetic stress in three commonly used transgenic mouse models of FAD. Assessment of mitochondrial motility, distribution, dynamics, morphology, and metabolomic profiling revealed the specific effect of each FAD mutation on the development of mitochondrial stress and dysfunction. Inhibition of mitochondrial trafficking was characteristic for embryonic neurons from mice expressing mutant human presenilin 1, PS1(M146L) and the double mutation of human amyloid precursor protein APP(Tg2576) and PS1(M146L) contributing to the increased susceptibility of neurons to excitotoxic cell death. Significant changes in mitochondrial morphology were detected in APP and APP/PS1 mice. All three FAD models demonstrated a loss of the integrity of synaptic mitochondria and energy production. Metabolomic profiling revealed mutation-specific changes in the levels of metabolites reflecting altered energy metabolism and mitochondrial dysfunction in brains of FAD mice. Metabolic biomarkers adequately reflected gender differences similar to that reported for AD patients and correlated well with the biomarkers currently used for diagnosis in humans.

**Conclusions:**

Mutation-specific alterations in mitochondrial dynamics, morphology and function in FAD mice occurred prior to the onset of memory and neurological phenotype and before the formation of amyloid deposits. Metabolomic signatures of mitochondrial stress and altered energy metabolism indicated alterations in nucleotide, Krebs cycle, energy transfer, carbohydrate, neurotransmitter, and amino acid metabolic pathways. Mitochondrial dysfunction, therefore, is an underlying event in AD progression, and FAD mouse models provide valuable tools to study early molecular mechanisms implicated in AD.

## Introduction

Alzheimer's Disease (AD) is a devastating neurodegenerative disorder characterized by progressive memory loss and impairment in behavior, language, and visuospatial skills [1]. The familial form of AD (FAD) has an early-onset and is caused by mutations in the amyloid precursor protein (APP) and presenilin 1 and 2 (PS1 and PS2) genes that lead to the accumulation of Aβ peptide [2]. Recent data suggest Aβ directly affects mitochondria early in AD contributing to the loss of synaptic function and plasticity, which are increasingly recognized as major mechanisms responsible for memory loss in AD [3]. Indeed, a decrease in cytochrome oxidase activity and energy metabolism and an increase in free radical production were detected in AD patients and AD mice prior to the formation of amyloid plaques and memory loss [4]–[7]. In neurons from AD mice, Aβ associates with mitochondrial membranes altering their trafficking, function and dynamics with synaptic mitochondria being particularly susceptible to Aβ-induced damage [8]–[12].

Mitochondria are dynamic organelles that actively move within the axons to ensure adequate energy supply. In the cell body, mitochondrial movement is essential for proper calcium buffering and energy transfer and distribution [13]–[16]. Therefore, it is not surprising that altered dynamics could be a causative factor in mitochondrial failure. However, the evaluation of the effect of particular FAD mutations on the development of mitochondrial dysfunction has not been done. Gaining such knowledge is important in order to identify the best animal models that most closely mimic human disease to reveal molecular mechanisms of mitochondrial dysfunction in AD, to develop the efficient tools for early diagnosis, and for the evaluation of the novel therapeutic approaches.

In the present study, we utilized three FAD transgenic mouse models, APP, PS1, and APP/PS1. In order to evaluate the impact of the particular mutation on mitochondrial dynamics and function, we examined organelle motility, distribution, ultrastructure and function in neurons and brain tissue of FAD mice starting from embryonic day 17 till the age when the onset of memory and the development of amyloid deposits become prominent for each particular mouse model. Thus, axonal trafficking was examined in embryonic neurons (E17); mitochondrial distribution and ultrastructure was evaluated in neurons (E17) and brain tissue of FAD mice 8, 12 and 40 weeks of age; brain function and metabolomic profiling was done in brain tissue from 16, 28 and 36 weeks old animals. We found that in all these mice mitochondrial dysfunction preceded the onset of memory phenotype and the formation of amyloid plaques, however, the development of mitochondrial abnormalities was mutation specific. Inhibition of axonal trafficking was the earliest dysfunction detected in embryonic neurons from PS1 and APP/PS1 mice. Loss of morphology was most prominent in APP and APP/PS1 mice. Application of metabolomic profiling allowed identifying metabolites and metabolic pathways that were affected in all three FAD mouse models, along with specific metabolomic signatures of mitochondrial stress associated with particular FAD mutation. Metabolic biomarkers adequately reflected gender differences similar to that reported for AD patients and correlated well with the biomarkers currently used for diagnosis in humans. Our data validate the use of FAD mice as a tool to study mitochondrial dysfunction, which underlies the development of AD in multiple FAD mouse models regardless of the origin of mutation and is accompanied by specific metabolic changes useful for early diagnosis and monitoring the disease progression.

## Results

### Trafficking of mitochondria is inhibited in hippocampal neurons from PS1 and APP/PS1 mice

**Figure 1:**
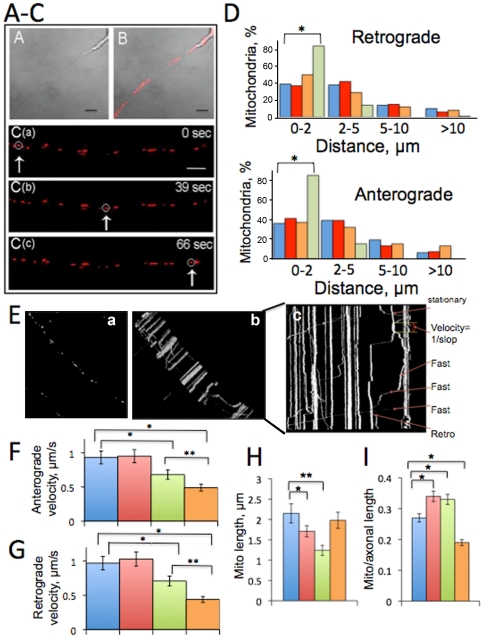
Mitochondrial trafficking and distribution in primary embryonic neurons from APP, PS1 and APP/PS1 mice. **A–C.** Real time imaging of mitochondrial movement within the axon of Hip neuron from PS1 mouse 7 days in culture. **A.** Phase image of the axon; cell body is in the upper right corner. **B.** Same axon with mitochondria visualized using TMRM. Scale bar, 10 µm. **C (a–c).** Recording of mitochondrial movement in live axon: arrow and circle indicate the progress of the same organelle along the axon with time. Images were acquired using LSM 510 laser scanning microscope (Carl Zeiss) with 100× oil DIC (1.4 na) lens. Scale bar, 5 µm. **D.** Mitochondria in PS1 neurons cover significantly shorter distances between stops in both, anterograde and retrograde directions compared to organelles in NTG, APP or APP/PS1 neurons. Almost no mitochondria in PS1 neurons cover distances longer than 10 µm. Number of organelles taken into analysis is the same as in (**F,G**). Blue – NTG; Red – APP; Orange – APP/PS1; Green – PS1. *p<0.001. **E.** Selective analysis of mitochondrial dynamics was done using analytical software (Analyze) that allows to trace each organelle from the first frame (**a**) through all 600 frames of the movie (stacked movie frames, **b**) to generate a final profile of movement (**c**). Resultant kymograph (**c**) is used to calculate velocities and identify the number of stationary and moving mitochondria. Rates of organelle motility in anterograde (**F**) and retrograde (**G**) directions include analysis of movement of 74 to 285 individual mitochondria in 24 to 33 neurons from at least three individual platings for each genotype. *, p<0.001; **, p<0.05. **H.** Length of 85–156 individual mitochondrion was estimated in five randomly selected axons in E17 neurons. *, p<0.001; **, p<0.0001. **I.** The number of organelles normalized per axonal length in embryonic neurons from NTG and AD mice used in axonal trafficking analysis. Number of mitochondria increases in APP and PS1 mice, and decreases in APP/PS1 cells comparing to NTG neurons. *, p<0.01. Colors as in **D**.

Altered mitochondrial motility, distribution and dynamics were shown to contribute to the development of AD in animal models and in humans [8], [17]–[22]. However, it is not clear whether different FAD mutations affect mitochondrial dynamics and function to the same extent and within the same time frame relevant to the development of AD. To determine the effect of FAD mutations on mitochondrial motility, we investigated axonal trafficking of mitochondria in primary hippocampal (Hip) and cortical (Ctx) neurons from three FAD transgenic mouse models. The first model includes mice that over-express mutant human APP gene. These mice are characterized by the presence of high levels of soluble Aβ by 6 months of age, and fibrillar plaque deposition and behavioral deficits that appear between 9 and 11 months of age [23]–[25]. The second model, where mice express mutant human PS1 (M146L), is characterized by increased levels of murine Aβ42. However, these mice do not form amyloid plaques and do not demonstrate cognitive impairment till at least 12 months of age [26], [27]. Transgenic mice from the third model express both mutant human APP and PS1 (APP/PS1) [28]. These mice have accelerated AD phenotype characterized by amyloid deposits and behavioral deficits in as little as 13–16 weeks [28]. We have examined axonal trafficking of mitochondria in live embryonic neurons from APP, PS1 and APP/PS1 mice, and compared it to control animals utilizing real time imaging [29]. Non-transgenic (NTG) littermates obtained from the crosses between APP and PS1 mice were used as controls similar to the previous study [28]. Mitochondria in neurons were visualized using the specific dye tetramethylrhodamine methyl ester (TMRM) that does not affect organelle motility [29] (Figure 1 A–C, Movie S1). For each neuronal genotype, we estimated rates of mitochondrial movement in anterograde (from the cell body) and retrograde (to the cell body) directions, distance each mitochondrion traveled between stops, the percent of stationary mitochondria, average mitochondrial length, and number of organelles per axonal length (Figure 1D–I).

We have found that FAD mutations have markedly different effects on mitochondrial dynamics. Thus, movement of mitochondria in PS1 and APP/PS1 neurons was significantly inhibited in both anterograde and retrograde directions comparing to NTG littermates. Anterograde motility in NTG neurons (0.93±0.55 µm/sec) was reduced in PS1 (0.68±0.33 µm/sec, *p*<0.001) and APP/PS1 neurons (0.49±0.29 µm/sec, *p*<0.001) (Figure 1F). Similarly, mitochondrial movement in retrograde directions changed from 0.97±0.63 µm/sec in NTG neurons to 0.71±0.33 µm/sec in PS1 (*p*<0.001) and 0.41±0.63 µm/sec in APP/PS1 neurons (*p*<0.001) (Figure 1G). Movement of mitochondria in APP/PS1 neurons was affected to a greater extent than in singly transgenic PS1 mice (Figure 1 F,G, *p*<0.05). However, trafficking of mitochondria in neurons from APP mice was not affected and neither the velocities nor the pattern of motion differ from NTG neurons (Figure 1D,F,G). Remarkably, mitochondria in PS1 neurons not only moved slowly but also tended to cover significantly shorter distances between stops (Figure 1D). Thus, 80% of mitochondria in PS1 neurons covered distances smaller than 2 µm comparing to 40% in NTG, APP and APP/PS1 neurons, and almost none of the organelles in the PS1 neurons covered distances greater than 5 µm in both directions (Figure 1D). Despite stronger inhibition of trafficking in APP/PS1 neurons compared to PS1 cells (Figure 1F,G), the distances covered by mitochondria in APP/PS1 neurons did not differ from NTG or APP cells (Figure 1D). We also analyzed the amount of stops per distance and determined that mitochondria in PS1 and APP/PS1 cells stopped more often. Thus, the ratio of stops per distance was 0.23 and 0.27 in NTG and APP neurons, respectively. However, in PS1 and APP/PS1 neurons, this number was significantly higher and reached 0.45 and 1.17, respectively. Along with reduced motility, mitochondria in PS1 and APP/PS1 neurons became progressively immobilized. In neurons from NTG and APP mice 58 to 60% of organelles was stationary. In PS1 and APP/PS1 neurons, the percent of immobilized mitochondria increased to 85 and 92, respectively (*p*<0.01). Analysis of mitochondrial length and distribution in axons of embryonic neurons revealed significant reduction in the number of organelles per axonal length in APP/PS1 neurons comparing to NTG, PS1 and APP cells (*p*<0.01) (Figure 1I). Mitochondria were significantly shorter in neurons from APP (1.71±1.17 µm, *p*<0.001) and PS1 (1.24±0.69 µm, *p*<0.0001) neurons compared to NTG (2.15±1.13 µm) and APP/PS1 (1.98±1.21 µm) cells (Figure 1H); however, their number per axonal length was significantly increased compared to NTG and APP/PS1 neurons (Figure 1I). Thus, our data suggest that mitochondrial trafficking and distribution are altered in embryonic neurons from FAD mice. Individual FAD mutations differentially affect mitochondrial dynamics thus suggesting distinct mechanisms. Synergistic effect of PS1 and APP mutations on axonal trafficking might explain the stronger phenotype observed in APP/PS1 neurons comparing to PS1.

### Inhibition of mitochondrial trafficking in PS1 and APP/PS1 mice does not correlate with Aβ levels

**Figure 2:**
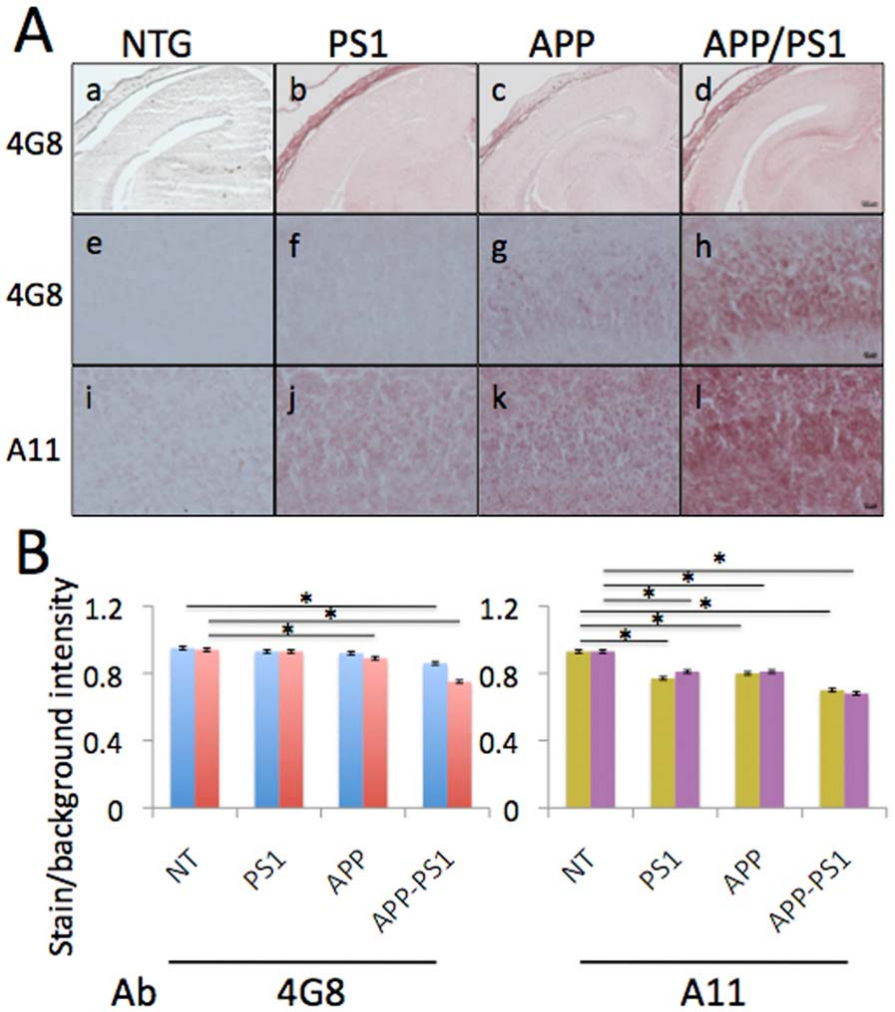
Levels of Aβ in brain tissue of 1-day-old APP, PS1, APP/PS1 and NTG mice. **A.** Levels of Aβ in brain tissue were determined using monoclonal antibody 4G8 that recognizes human and mouse abnormally processed Aβ isoforms and precursor forms (**a–d**). Scale bar, 200 µm. Levels of Aβ in CA1 Hip pyramidal neurons detected with 4G8 antibody (**e–h**) or antibody A11 that recognizes Aβ oligomers (**i–l**). Scale bar, 20 µm. **B.** Densitometry measurements of immunostaining produced by 4G8 and A11 antibodies in a–l. The lighter staining of the NTG brain resulted in ratios closer to 1, indicating the stained tissue was almost as bright as the bare slide and therefore, had lower Aβ immunoreactivity. As the Aβ immunoreactivity increases, the normalized intensity ratios decrease, with the lowest values observed in the APP/PS1 mouse brain because the tissue becomes darker and closer to black. Data is expressed as mean ± SEM. Blue – Ctx 4G8; red – Hip 4G8; green – Ctx A11; purple – Hip A11. * <0.01. Ab – antibody.

We next determined whether differences observed in the extent of mitochondrial trafficking inhibition in FAD mice depended on the levels of Aβ. Estimation of Aβ levels in the brain tissue of the newborn mice has not been done before. To specifically compare levels of Aβ in the Ctx and Hip tissue of the FAD newborn mice, we applied a well-established immunohistochemistry technique [25] using a panel of anti-Aβ antibodies (Figure 2). Detection with a 4G8 monoclonal antibody that recognizes both, murine and human abnormally processed Aβ isoforms along with the precursor forms of Aβ revealed lack of plaques in any FAD mouse brains examined (Figure 2 a–d). Levels of Aβ in the CA1 region of the Hip were very low in PS1 mice compared to APP and APP/PS1 animals (Figure 2A, f–h). Levels of Aβ in CA1 region in APP/PS1 mice were dramatically increased compared to APP mice (Figure 2A, g, h). Similar increase in Aβ levels in APP/PS1 mice compared to APP was observed using 6E10 antibody that recognizes only human Aβ (data not shown). Moreover, application of polyclonal A11 antibody that specifically recognizes murine and human Aβ oligomers revealed abundant presence of these species in all three FAD mouse brains (Figure 2A, i–l). The densitometry measurements confirmed the increase of Aβ levels in APP and APP/PS1 mice specifically in the Hip as measured with 4G8 antibody (Figure 2B). The increase in the levels of Aβ oligomers was significant in both Ctx and Hip regions in PS1, APP and APP/PS1 mice (Figure 2 B). The lack of correlation between the extent of trafficking inhibition and levels of Aβ in PS1 and APP/PS1 mice suggests different mechanisms.

### Inhibition of axonal trafficking is a general defect that occurs in all three FAD mouse models and is not specific for mitochondria

**Figure 3:**
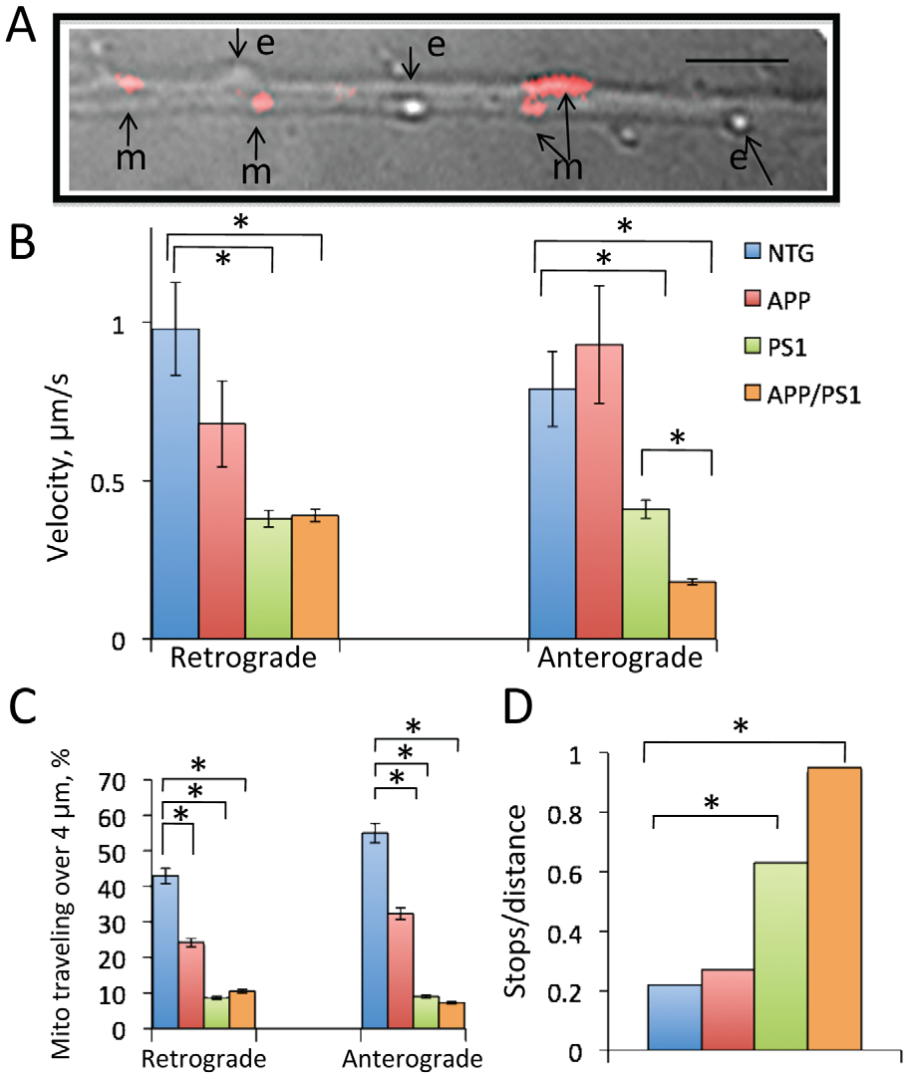
Inhibition of axonal trafficking in neurons from PS1 and APP/PS1 mice is not specific to mitochondria. **A.** Analysis of real time movement of round-shaped vesicles (endosomes or lysosomes, “v”) that were not stained with TMRM was done using same movies that were acquired to study mitochondrial (‘m’) trafficking. Scale bar, 4 µm. **B.** Similar to mitochondria, round-shaped vesicles move with reduced velocities in both, anterograde and retrograde directions, travel shorter distances between stops in neurons from APP, PS1 and APP/PS1 mice (**C**), and stop more frequently in neurons from PS1 and APP/PS1 mice (**D**). Analysis was done in randomly selected neurons (13–19 for every genotype); pattern and rate of motion of 10 to 58 individual vesicles was analyzed. *p<0.001.

We next examined whether inhibition of axonal trafficking in FAD mice was specific for mitochondria. Using the same movies that were generated to study mitochondrial motility, we analyzed axonal trafficking of small round-shaped vesicles that were not stained with the mitochondrial marker TMRM and which motility was traceable in the bright field recording (Figure 3A, “e”). Based on their size and appearance, these vesicles most likely are endosomes or lysosomes [29]. Similar to the results obtained for mitochondrial movement, we have determined that round vesicles moved slower in both anterograde and retrograde directions (Figure 3B), covered shorter distances between stops (Figure 3C), and stopped more frequently in neurons from PS1 and APP/PS1 mice (Figure 3D). However, contrary to the movement of mitochondria, specific and significant inhibition of retrograde transport of round vesicles was also observed in neurons from APP mice (Figure 3B). Thus, rates of retrograde transport in APP neurons (0.68±0.31 µm/s) were significantly reduced comparing to the rates in NTG neurons (0.98±0.23 µm/s, *p*<0.001). Vesicles in APP neurons also covered shorter distances comparing to NTG neurons. In NTG cells, 42% and 55% of vesicles covered distances longer than 4 µm in retrograde and anterograde directions, respectively (Figure 3C). However, in APP neurons, the number of vesicles that cover distances over 4 µm dropped to 24 and 32%, respectively (*p*<0.01). In PS1 and APP/PS1 cells, the number of vesicles that cover 4 µm was reduced to 10% (Figure 3C). The ratio between stops per distance was significantly increased in PS1 (0.63, *p*<0.001) and APP/PS1 (0.95, *p*<0.001) neurons compared to NTG (0.22) or APP (0.27) neurons (Figure 3D). Our data suggest inhibition of axonal trafficking in embryonic PS1, APP and APP/PS1 neurons is a general defect that affects movement of multiple vesicles and organelles.

### Neurons with inhibited mitochondrial trafficking are more susceptible to excitotoxic cell death

Besides energy production, regulation of intracellular calcium is another main function of mitochondria. Mitochondrial motility and positioning in neurons is essential for proper calcium buffering [13]–[15]. Since we found that axonal trafficking of mitochondria was significantly altered in neurons from APP/PS1 and PS1 mice, we investigated whether these cells exhibit increased sensitivity to calcium entry caused by stimulation of NMDA receptors.

**Figure 4:**
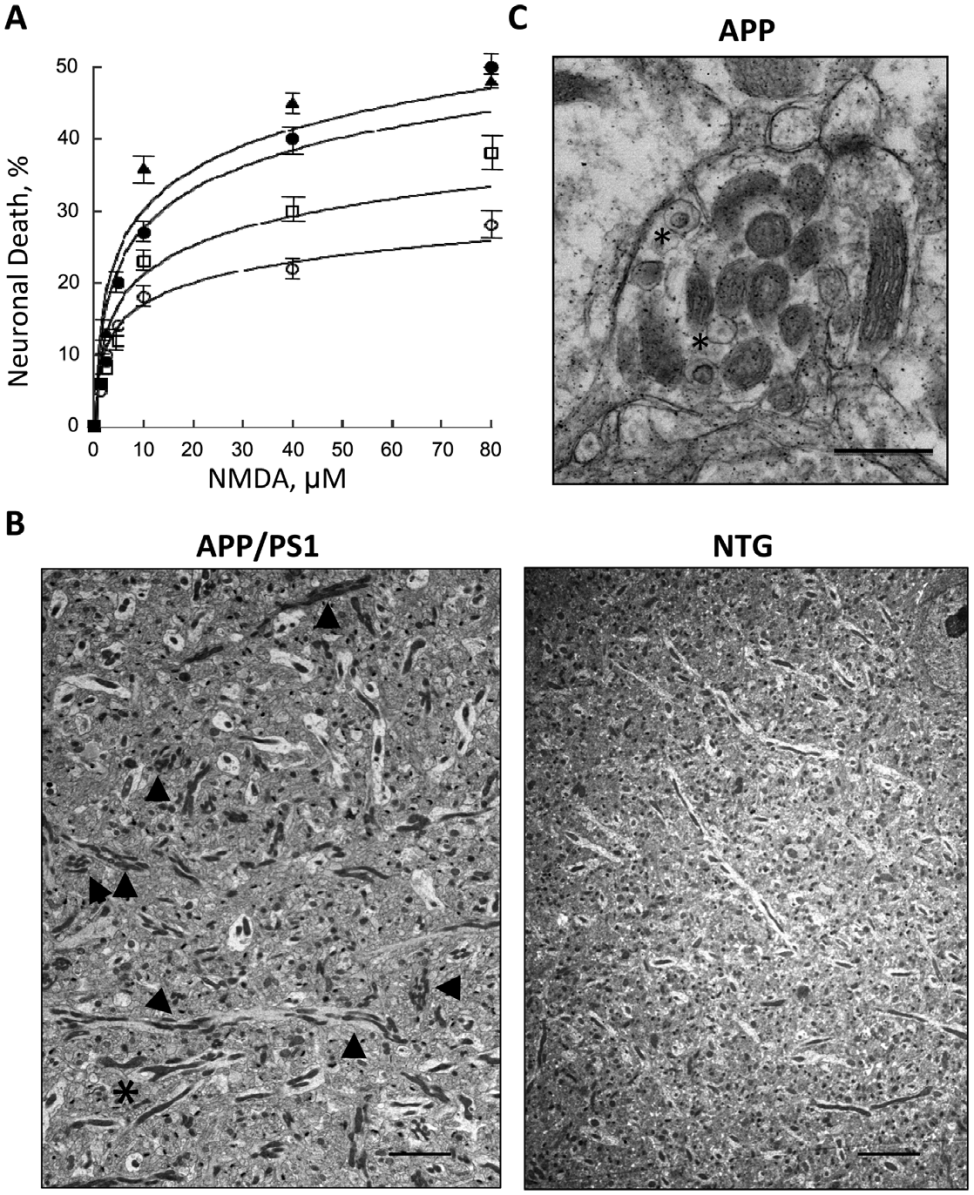
Mitochondrial distribution is altered in hippocampus of AD mice. **A.** Neurons in APP/PS1 and PS1 mice exhibit increased sensitivity to NMDA treatment. Open circles – NTG; Open squares – APP; Close circles – APP/PS1; Triangles – PS1. **B.** Electron micrographs of an altered mitochondrial distribution in brain of APP/PS1 mouse 8 weeks old compared to NTG mouse of the same age. Asterisk denotes mitochondria with altered shape; arrowheads denote accumulation of mitochondria in neuropils. Scale bar, 5 µm (APP/PS1), 2 µm (NTG). **C.** Accumulation of normal and degenerating (asterisks) mitochondria in brain of APP mouse 12 weeks old. Scale bar, 500 nm.

Indeed, treatment with different doses of NMDA caused excitotoxic cell death in neurons from all three FAD mouse models that correlated with the extent of mitochondrial trafficking inhibition (Figure 4A). Treatment with 80 µM of NMDA caused 25% loss of neurons from NTG and 30% from APP mice (Figure 4A, open circles and squares). However, neurons from PS1 and APP/PS1 demonstrated 45% and 50% cell loss, respectively (Figure 4A, closed circles and triangles). The response to the lower doses of NMDA was also more robust in APP/PS1 and PS1 neurons compared to NTG or APP neurons (Figure 4A, doses below 10 µM). Our data suggest that neurons with inhibited mitochondrial motility become susceptible to excitotoxic cell death.

### Alterations in mitochondrial morphology and localization in FAD mice precede the onset of memory deficit and formation of amyloid plaques

**Figure 5:**
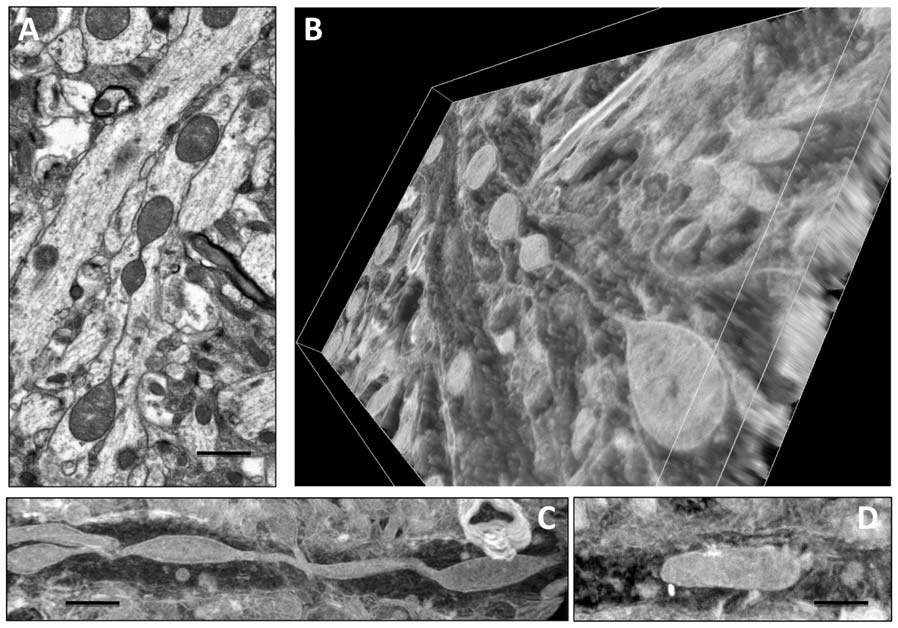
Mitochondria in APP and APP/PS1 mouse brains acquire abnormal shape. **A.** 2D EM micrograph of mitochondrion with abnormal shape in Hip tissue of APP mouse 40 weeks of age. **B.** 3D reconstruction of ten serial sections of consecutive EM micrographs of the same tissue as in (**A**). **C.** 3D reconstruction of mitochondrial structure in Hip tissue of APP/PS1 mouse 24 weeks of age. Note the dramatic elongation of mitochondrion in APP/PS1 tissue compared to the length and shape of the organelle in the brain of NTG mouse of the same age (**D**). Scale bar, 1 µm.

Mitochondrial distribution is essential for maintaining synaptic function and transmission [20], [30]. Mislocalization of mitochondria induced by Aβ42 in *Drosophila* was sufficient to cause late-onset behavioral deficits [31]. Similarly, the degree of cognitive impairment in AD transgenic mice has been linked to the extent of synaptic mitochondrial dysfunction [32]. Therefore, we examined whether synaptic and non-synaptic mitochondrial structure, integrity and distribution were altered in Hip tissue of APP, PS1, and APP/PS1 mice 8, 12, 24, 30 and 40 weeks of age using electron microscopy (EM). We have found that Mito distribution was already altered in neuropils in all three FAD mice between 8 and 12 weeks of age (Table 1). “Piling” of mitochondria in neuropils in APP/PS1 mice and trafficking “jams” consisted of normal and degenerating mitochondria were observed in brain tissue from all three FAD mice (Figure 4B, C). However, quantification of mitochondrial distribution revealed a reduction in the number of mitochondria per neuropil or per the length of neuropil in all three FAD mice starting at 8–12 weeks of age and persisting in APP/PS1 mice till 40 weeks of age (Table 1). Neuropils from PS1 and APP/PS1 mice had the most pronounced reduction in the number of organelles at 12 weeks. This is consistent with our observations that the number of Mito was significantly reduced in the embryonic APP/PS1 neurons suggesting that altered mitochondrial distribution in these mice is associated with the early stages of AD progression (Figure 1F). Interestingly, mitochondrial length was significantly increased in the brains of FAD mice with reduced number of mitochondria per neuropil (Table 1, Figure 5A–C).

**Table 1:** Mitochondrial distribution and structural characteristics in brain tissue from NTG, APP, PS1, and APP/PS1 mice based on electron microscopy examination.

Genotype	Age, weeks	# of Neuropils	Neuropil length, µm	# of Mito	Mito per neuropil	Mito per neuropil length	Mito length, µm	Abnormal Mito, %
**NTG**	8	83	290.5	143	1.7	0.49	1.11±0.96	0
	12	14	150.6	53	3.8	0.35	2.11±0.89	0
	40	25	239.6	62	2.5	0.26	2.31±1.05	4
**APP**	8	29	405.3	58	2.0	0.14*	2.03±1.71*	3
	12	16	151.9	48	3.0	0.32	2.85±1.72	5
	40	21	158.5	39	1.9	0.25	2.01±1.65	20
**PS1**	8	35	200.9	74	2.1	0.37	1.27±1.01	2
	12	21	231.6	40	1.9	0.17*	4.06±3.12**	1
	40	15	133.4	44	2.9	0.33	2.32±1.42	5
**APP/PS1**	8	60	407.2	114	1.9	0.28*	1.23±0.98	1
	12	23	180.4	41	1.8	0.23*	2.85±1.42*	7
	40	23	407.7	49	2.1	0.12*	3.31±2.89**	30

*P<0.001 and **P<0.0001 vs. 8, 12, and 40 weeks old NTG mice. Mito: mitochondria.

**Figure 6:**
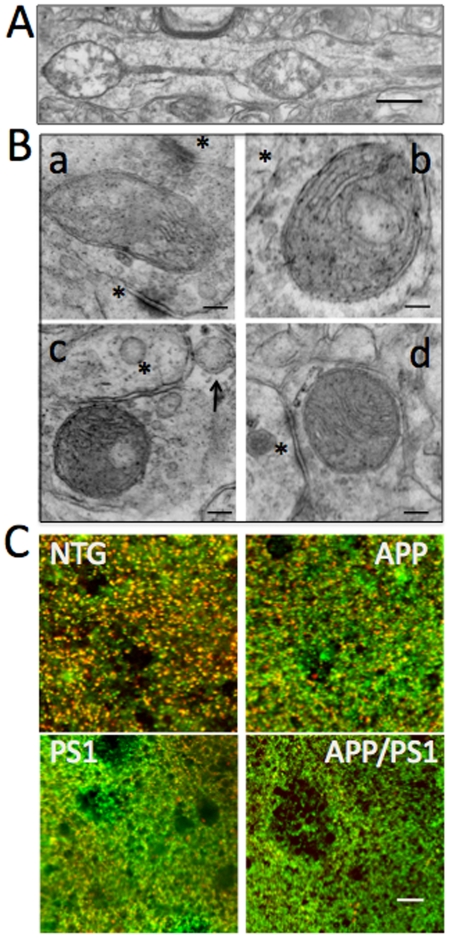
Mitochondria in AD mice have altered morphology and reduced oxidative activity. **A.** Progressive age-dependent accumulation of abnormal mitochondria in AD mouse brains with dramatic loss of cristae integrity. Mitochondrion in APP mouse 45 weeks of age is shown. Scale bar, 1 µm. **B.** Abnormal synaptic mitochondria in APP/PS1 mouse brains were already observed at 8 weeks of age (a–c). Arrow denotes swollen mitochondria with total loss of inner structure. (d) Mitochondria in NTG mouse of the same age. Asterisks indicate the synapses; scale bar, 100 nm. **C.** Loss of mitochondrial oxidative activity in relationship to total mitochondrial mass in Hip live brain slices detected using MTG and MTO. Mitochondria in NTG brain have extensive colocalization of green (MTG, mitochondrial mass) and red (MTO, oxidative activity) fluorescence with the ratio of MTG/MTO = 0.96. Mitochondria in PS1, APP and APP/PS1 mouse brains have reduced oxidative activity as judged by the loss of red fluorescence intensity. The ratios of MTG/MTO were estimated to be: 0.8 (PS1), 0.6 (APP), and 0.5 (APP/PS1). Images were acquired using LSM 510 with 40× lens. Scale bar, 10 µm.

Detailed examination of the mitochondrial morphology revealed the presence of significant structural abnormalities in APP/PS1 and APP mice starting at 8 and 12 weeks of age, respectively, and reaching 20–30% by the age of 40 weeks (Figure 5A–C, Movie S2, Table 1). Mitochondria acquired abnormal shape, which consisted of very narrow membranous segments alternated with swollen round-shaped areas with perturbed cristae resembling “beads-on-the-string” (Figure 5A–C, Movie S2). Three-dimensional reconstruction of the EM micrographs demonstrated the age-dependent increase in the presence of these abnormal organelles in the neuropils of APP and APP/PS1 mice while only 4 to 5% of similar structures were found in the brain of PS1 and NTG mice 40 weeks of age (Table 1). Abnormal mitochondria significantly increased in length reaching in some cases 26–30 µm (Movie S2). These organelles were characterized by complete loss of cristae (Figure 6A). Since narrow segments of abnormal mitochondria are very thin, accurate estimation of the organelle length in AD mice requires 3D reconstruction of multiple consecutive micrographs. Therefore, the estimation of mitochondrial length based on 2D micrographs (Table 1) may underestimate the extent of organelle elongation in “beads-on-the-string” structures and could convolute the real increase in mitochondrial length in AD mice with age.

Examination of the ultrastructure of synaptic mitochondria also revealed the presence of the degenerating organelles with altered cristae organization (Figure 6B, a–c). Abnormal synaptic mitochondria were detected in all three FAD mice starting at 8–12 weeks of age. Thus, changes in organelle structure and morphology occur in all three FAD mouse models prior to the onset of memory phenotype and plaque formation [23]–[25].

### Mitochondrial activity is reduced in brain slices from APP, PS1 and APP/PS1 mice

Next we examined whether mitochondrial structural abnormalities observed in brains of APP, PS1 and APP/PS1 animals were associated with the inhibition of mitochondrial function. We adapted the method that allowed measuring mitochondrial mass and activity in brain slices from control and FAD mice [33], [34]. This approach utilizes application of two mitochondrial specific probes, MitoTracker Green (MTG), which becomes fluorescent when it accumulates in the mitochondrial lipid environment regardless of membrane potential, and MitoTracker Orange (MTO), which measures mitochondrial oxidative activity. MTO becomes fluorescent only when oxidized with molecular oxygen in actively respiring mitochondria. The use of both probes avoids isolation and purification of mitochondria thus preserving cell and tissue integrity. The ratio between MTO and MTG fluorescence intensities determines the fraction of active mitochondria in total organelle content. Brains from NTG, APP, APP/PS1 and PS1 mice 7 months old were cut into 50 µm-thick slices. Each brain slice was incubated in either buffer alone or in buffer containing MTG and MTO. Tissue slices were washed and imaged using LSM 510 confocal microscope. We have found that mitochondria in all three FAD mice lost oxidative activity comparing to NTG mice (Figure 6C). The decrease was most prominent in the brains of APP and APP/PS1 mice (loss of ∼50%). The decrease of oxidative activity in PS1 mice was about 35%. Thus, changes in mitochondrial ultrastructure observed in FAD mice (Figure 5, 6) correlate with the loss of mitochondrial function.

### Expression of fission and fusion proteins is not altered in APP, PS1 and APP/PS1 mice

**Figure 7:**
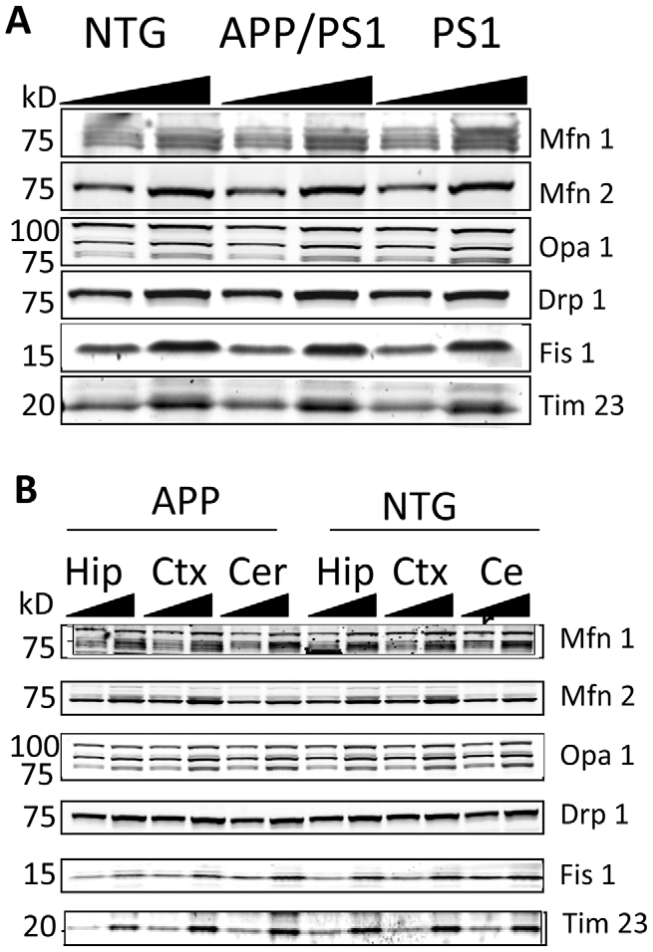
Expression of mitochondrial fusion and fission proteins is not altered in brain tissue from AD mice. Representative immunoblot (**A**) revealed no differences in expression of fusion and fission proteins in brain tissue from 12 months old APP/PS1, PS1 and NTG mice. **B.** No changes in expression of fission/fusion proteins were found in different brain regions (hippocampus, Hip, cortex, Ctx, and cerebellum, Cer) in APP mouse 13 months old compared to NTG mouse of the same age. Each sample was loaded twice with second sample having 2× concentration.

Alterations in mitochondrial dynamics, fission and fusion in particular, have been implicated in AD progression in humans [9], [30], [35], [36]. Therefore, we examined whether changes in mitochondrial morphology observed in the brains of FAD mice were related to the altered expression of key proteins involved in mitochondrial dynamics. First, we investigated whether levels of expression of mitochondrial fusion (Opa1, Mfn1, and Mfn2) and fission proteins (Drp1 and Fis1) were altered in the whole brain tissue extracts from NTG, APP/PS1 and PS1 mice 40 weeks of age (Figure 7A). As a control, we used the essential component of the inner mitochondrial membrane translocase complex Tim 23. Immunoblot analysis revealed no changes in the levels of expression of all six proteins. No significant differences in overall mitochondrial content were noted between samples from APP/PS1, PS1 and NTG mice evident by the constant expression levels of Tim 23, a mitochondrial marker. We next determined levels of expression of fission/fusion proteins in the different brain regions in APP and NTG mice 45 weeks of age (Figure 7B). Immunoblot analysis of protein extracts from Hip, Ctx and cerebellum (Cer) also revealed no changes in the expression levels of all proteins examined (Figure 7B). Our data suggest that changes in mitochondrial morphology observed in APP and APP/PS1 mice (Figure 5, 6) were not caused by altered expression of mitochondrial fission or fusion proteins.

### APP, PS1 and APP/PS1 mice have distinct gender- and mutation- related changes in metabolomic profiles in brain tissue

**Figure 8:**
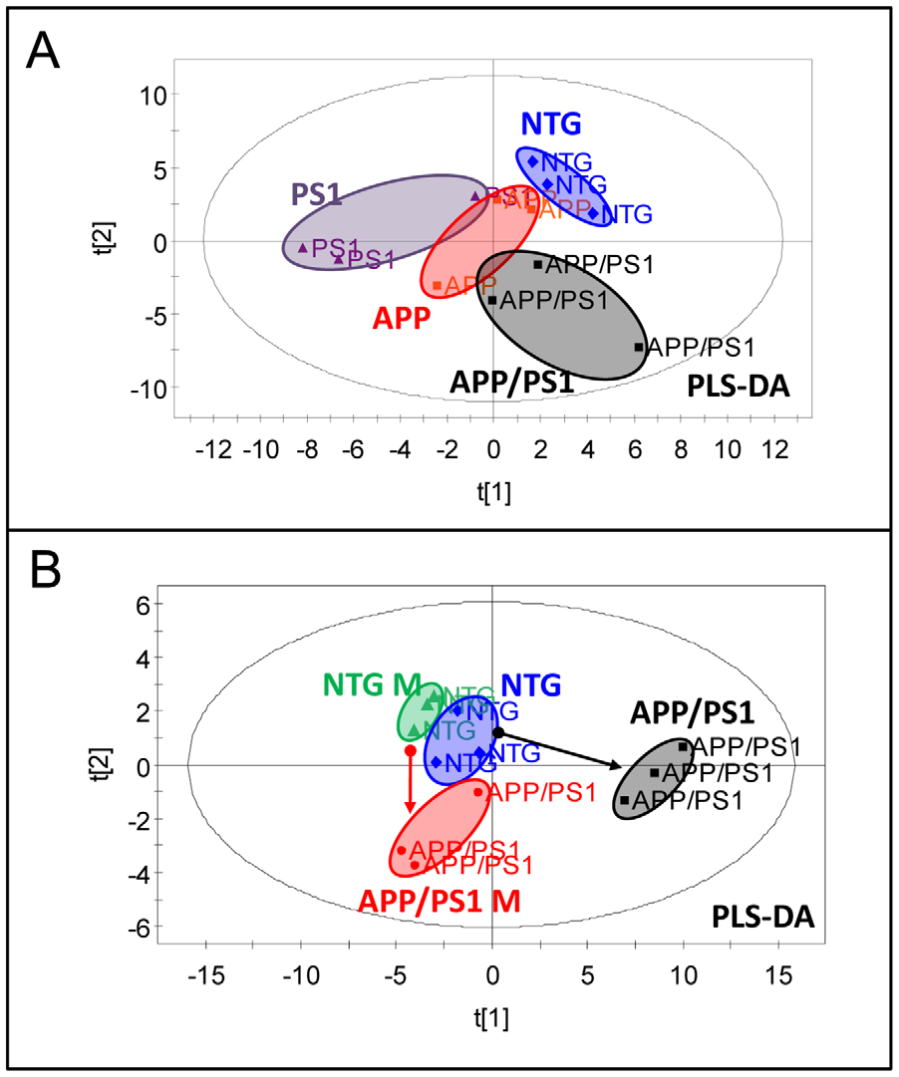
Brain tissue of APP, PS1, and APP/PS1 mice has distinct metabolomic profiles compared to NTG mice. **A.** PLS-DA score plot showing distinct metabolomic profiles of Hip brain tissue from PS1, APP and APP/PS1 mice compared to NTG mice. **B.** PLS-DA score plot demonstrating a significant gender effect on metabolomic profiles in APP/PS1 mice. Metabolomic alterations associated with mitochondrial dysfunction were more pronounced in female than in male APP/PS1 mice. Note significant differences in metabolomic profiles between APP/PS1 males and females and much smaller variation between NTG males and females. Each group included 3 mice 16 week of age.

**Figure 9:**
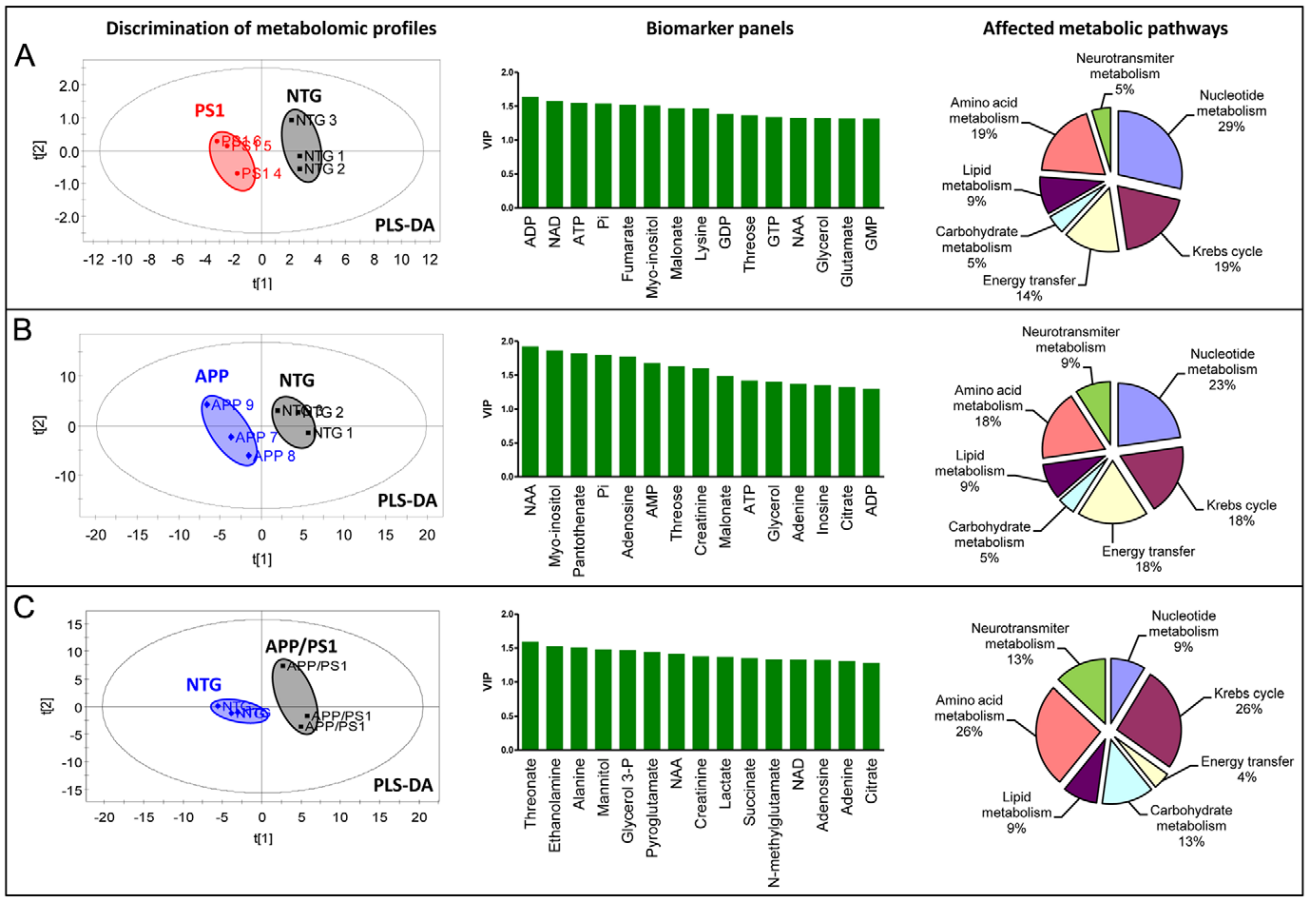
Comparison of individualized metabolomic profiles and affected metabolic pathways in FAD mouse models. **A, C and E.** PLS-DA score plots showing distinct metabolomic profiles of PS1 (**A**), APP (**B**) and APP/PS1 (**C**) female mice compared to NTG littermates. **A, B and C, middle panels.** Panels of specific biomarkers as a plot of variable importance in the projection (VIP) indicating the 15 most significant metabolites in discriminating between metabolomic profiles of NTG and Tg groups in the PLS-DA model. **A, B and C, right panels.** Metabolic pathways specifically affected in each FAD mouse model. APP and PS1 mice were 36 weeks old, APP/PS1 – 16 weeks old.

We have demonstrated that mitochondrial trafficking, distribution, morphology and function are affected in brain tissue from all three FAD mice prior to the onset of cognitive decline or plaque formation. However, the development of mitochondrial abnormalities was different in each FAD mouse model. Thus, inhibition of mitochondrial trafficking was observed in embryonic neurons from PS1 and APP/PS1 mice, but not in APP mice. Similarly, changes in mitochondrial morphology were prominent in APP and APP/PS1, but not in PS1 mice. To determine how particular FAD mutation affects metabolic pathways involved in mitochondrial function and energy metabolism, we analyzed metabolomic profiles in the Hip tissue of PS1, APP and APP/PS1 mice (Figures 8, 9, Table 2). Tissue from 36 week old APP and PS1 mice and 16 week old APP/PS1 mice was analyzed and compared to age- and gender-matched NTG littermates. The selection of mouse age for metabolomics was determined by differences between the onset of memory phenotype and amyloid plaques in FAD mice. Data analyses using PLS-DA revealed that PS1, APP and APP/PS1 mice have metabolomic phenotypes that are distinct from NTG littermates and also from each other (Figure 8A). Separate analysis comparing metabolomic signatures in APP/PS1 mice revealed significant gender-related differences (Figure 8B). Thus, NTG female and male mice have very similar metabolomic profiles, while profiles of APP/PS1 female and male mice differed significantly (Figure 8B). Additionally, the extent of alterations in metabolic signatures was greater in female APP/PS1 mice compared to male mice (Figure 8B, arrows between NTG and APP/PS1 males and NTG and APP/PS1 females).

**Table 2:** Relative changes of 30 most important metabolites in APP, PS1 and APP/PS1 transgenic mice compared to aged and gender matched NTG littermates.

PS1 mice	FC	VIP	p	APP mice	FC	VIP	p	APP/PS1 mice	FC	VIP	p
**ADP**	−0.56	1.59	0.003	**NAA**	−3.76	1.83	0.004	**Threonic acid**	1.08	1.59	0.003
**NAD**	−0.49	1.53	0.006	**Myo-inositol**	−0.50	1.76	0.006	**Ethanolamine**	1.67	1.52	0.012
**ATP**	−0.50	1.50	0.011	**Pantothenic acid**	−1.74	1.71	0.011	**Alanine**	2.22	1.51	0.048
**Pi**	−0.92	1.49	0.016	**Pi**	−0.93	1.69	0.014	**Mannitol**	1.22	1.48	0.024
**Fumaric acid**	−0.88	1.47	0.014	**Adenosine**	0.60	1.69	0.031	**Glycerol 3-P**	1.46	1.46	0.020
**Myo-inositol**	−0.55	1.46	0.016	**AMP**	1.60	1.58	0.041	**Pyroglutamic acid**	0.54	1.44	0.026
**Malonic acid**	−0.50	1.42	0.024	**Threose**	−0.16	1.54	0.047	**NAA**	0.65	1.41	0.032
**Lysine**	−0.65	1.41	0.030	**Creatinine**	−0.78	1.51	0.054	**Creatinine**	0.62	1.37	0.062
**GDP**	−0.41	1.34	0.044	**IMP**	−0.76	1.43	0.078	**Lactic acid**	0.50	1.37	0.044
**Threose**	−0.81	1.32	0.052	**Malonic acid**	−0.29	1.40	0.088	**Succinic acid**	0.88	1.35	0.054
**Glycerol**	−0.83	1.30	0.082	**ATP**	−0.63	1.33	0.114	**Methylglutamate**	0.69	1.33	0.143
**GTP**	−0.35	1.29	0.061	**Glycerol**	−0.42	1.32	0.123	**NAD**	0.22	1.33	0.057
**NAA**	−0.42	1.28	0.067	**Adenine**	0.50	1.28	0.135	**Adenosine**	1.39	1.32	0.063
**Glutamic acid**	−0.96	1.28	0.072	**Fumaric acid**	0.21	1.27	0.412	**Adenine**	0.70	1.30	0.065
**GMP**	−0.30	1.27	0.078	**Inosine**	−0.61	1.27	0.138	**Citric acid**	0.69	1.28	0.073
**Malic acid**	−0.55	1.27	0.076	**Citric acid**	−0.31	1.25	0.152	**Inosine**	1.65	1.24	0.090
**Creatinine**	−0.73	1.25	0.074	**ADP**	−0.46	1.23	0.181	**Glycine**	1.75	1.23	0.095
**Citric acid**	−1.64	1.21	0.110	**Beta- alanine**	0.28	1.20	0.175	**Aspartic acid**	1.42	1.23	0.099
**4-guanidinobutyrate**	−0.58	1.18	0.114	**Lactic acid**	−0.28	1.12	0.216	**Glyceric acid**	1.33	1.20	0.107
**AMP**	−0.39	1.16	0.115	**Nicotinic acid**	0.79	1.09	0.318	**Glutamine**	2.21	1.18	0.155
**IMP**	−0.28	1.16	0.158	**Lyxose**	−0.25	1.06	0.299	**Lysine**	2.25	1.16	0.122
**Beta- alanine**	−0.57	1.16	0.124	**Uracil**	−0.28	1.00	0.378	**THBA**	0.95	1.14	0.181
**Palmitic acid**	0.43	1.15	0.134	**Pyrophosphate**	0.48	0.97	0.356	**Uracil**	0.54	1.12	0.143
**Urea**	1.28	1.13	0.355	**GDP/NADP**	−0.21	0.96	0.413	**4-Aminobutyric acid**	1.94	1.09	0.165
**Cholesterol**	−2.57	1.05	0.258	**O-phosphocolamine**	0.30	0.96	0.395	**Cytidine/Hypoxanth**	0.81	1.02	0.195
**Succinic acid**	−0.58	1.04	0.179	**Methylglutamate**	−0.39	0.92	0.325	**Glycolic acid**	0.82	1.02	0.205
**Stearic acid**	0.46	1.03	0.194	**Glutamic acid**	−0.35	0.91	0.359	**Serine**	2.43	1.01	0.258
**Glycolic acid**	−0.26	1.00	0.201	**GTP**	−0.25	0.89	0.413	**IMP**	0.52	1.01	0.234
**Adenosine**	0.42	0.99	0.200	**Stearic acid**	0.55	0.89	0.341	**Uridine**	0.94	0.97	0.228
**Uracil**	−0.50	0.98	0.224	**Iminodiacetic acid**	0.60	0.89	0.410	**GMP**	0.53	0.95	0.246

For each group, the relative values of each metabolite (mean±SD) are the average obtained from three mice. The average basal metabolite values of NTG group were arbitrarily set at 1 for each group. The value of fold change (FC, log2 of fold change) for each metabolite is relative to the value in aged and gender matched NTG mice. Metabolites were selected based on VIP values; p values were estimated using Student's t-test.

Since gender was found to have a significant impact on metabolomic profiles, we focused on the comparison of the changes in metabolites specifically in the brain tissue of age-matched female APP, APP/PS1, PS1 mice and NTG littermates (Figure 9, Table 2). Metabolites in the separate pair comparison revealed presence of characteristic signatures of mitochondrial toxicity with altered tissue levels of energy metabolites ATP, ADP, AMP, nicotinamide adenine dinucleotide (NAD), adenosine, fumaric acid, adenine, creatine and β-alanine (Figure 9, middle panels). Increased levels of adenosine, AMP and fumaric acid and decreased levels of N-acetyl aspartate (NAA) strongly suggest presence of mitochondrial stress and energetic dysfunction. Metabolic pathway analyses revealed that in all three FAD mouse models there are significant alterations in the levels of metabolites involved in energy metabolism including nucleotide metabolism, mitochondrial Krebs cycle, energy transfer, carbohydrate, neurotransmitter and amino acid metabolic pathways (Figure 9, right panels). However, along with the pathways equally affected in all three FAD mouse models, we identified metabolic pathways and metabolites that were specific to the mutation. Thus, alteration in neurotransmitter metabolism and energy transfer pathway was affected to a greater extent in APP and PS1 mice (Figure 9, right panels). Synergistic effect of both mutations in APP/PS1 mice resulted in significantly stronger alterations in glycolytic pathway that involved Krebs cycle, and neurotransmitter and amino acid metabolism (Figure 9, right panels, Table 2). Metabolites that were uniquely affected in APP mice included panthotenic acid while threonate and ethanolamine were uniquely affected in APP/PS1 mice (Table 2). Moreover, in all three FAD mouse models we also found changes in the levels of NAA, myo-Inositol and creatinine (Figure 9, middle panel), the biomarkers that are currently used for the diagnosis of mild cognitive impairment and AD in humans [37]. Our data demonstrate that mitochondrial dysfunction is present early in development of AD in all three FAD mouse models regardless of the origin of the mutation. Moreover, metabolomic profiling could discriminate between the effect of gender and specific mutations on the metabolic pathways involved in mitochondrial dysfunction and energy failure.

## Discussion

Identification of the early molecular mechanisms underlying AD is of great importance in order to develop efficient methods for diagnosis and therapeutic intervention. Growing evidence suggests that mitochondrial dysfunction occurs prior to the onset of memory phenotype and plaque formation and is an important factor that modulates AD pathophysiology [38], [39]. Indeed, mitochondrial bioenergetics deficits precede the onset of AD symptoms in multiple AD animal models [40], [41]. In humans, regional glucose hypometabolism is detected many years before the disease onset in PS1 mutation carriers [42]. Biochemical studies demonstrate alterations in the activity of mitochondrial enzymes involved in Krebs cycle and electron transport chains in the post mortem AD brains [4], [43]. Recently, defects in mitochondrial morphology, distribution and dynamics have been found in the human AD brain tissue and in cellular and animal models of AD [20], [22], [36], [44]–[46]. However, the molecular mechanisms underlying mitochondrial dysfunction in AD remain elusive. Part of the problem relates to the lack of a model organism that recapitulates all aspects and complexity of AD, and specific limitations of the multiple animal models currently used in research laboratories.

We utilized three FAD mouse models to determine whether mitochondrial dysfunction was implicated in the early stages of AD regardless of the origin of FAD mutation, which would validate mitochondria as the target for early therapeutic intervention. We have found that inhibition of axonal trafficking of mitochondria was among the earliest abnormalities already detected in embryonic neurons from PS1 and APP/PS1 mice months before the onset of memory phenotype or formation of amyloid deposits [25], [28]. Previous studies demonstrated the direct effect of Aβ peptides on mitochondrial motility [18], [19], [31], [47], [48]. However, comparison of Aβ levels in the Hip tissue of newborn FAD mice with and without trafficking defect suggests that inhibition of mitochondrial trafficking in PS1 mice occurs through Aβ-independent mechanism. Indeed, it has been shown that mutant PS1 could directly affect axonal machinery by modulating the activity of glycogen synthase kinase-3β (GSK-3β) that controls the release of kinesin motor protein from the cargo [17], [21], [49]. In addition, PS1 mutations may affect mitochondrial trafficking by altering calcium homeostasis [50], [51]. Elevated calcium levels caused by PS1 mutations lead to an increased calcium uptake by mitochondria resulting in trafficking inhibition [15], [52]. However, the exacerbated trafficking phenotype observed in double mutant APP/PS1 mice where levels of Aβ were significantly elevated in Hip and Ctx tissue of newborn mice suggests synergistic effect of each mutation and implication of Aβ-dependent and independent mechanisms. Our data also support recent observations suggesting that Aβ oligomers represent toxic species that disrupt axonal trafficking [8], [53], [54]. The extent of trafficking inhibition in APP/PS1 mice correlated with significantly elevated levels of oligomeric Aβ in the Hip brain tissue of the newborn mice. Surprisingly, mitochondrial trafficking was not altered in embryonic neurons from APP mice where levels of Aβ were higher that in PS1 mice. However, since we have found that axonal trafficking of other cargo was inhibited in APP neurons, it is possible that levels of Aβ in embryonic neurons from APP mice did not yet reach the threshold required to cause detectable trafficking inhibition of mitochondria. This is supported by the observations that Aβ levels increase with time in the brain of the APP mice [23], [28], and that axonal trafficking inhibition of cargo other than mitochondria was detected in APP mice *in vitro* and *in vivo* [55], [56]. It remains to be determined whether inhibition of mitochondrial motility occurs in APP mice and whether that contributes to mitochondrial dysfunction.

Another important function of mitochondria in the cell includes buffering of intracellular cytosolic Ca^2+^ [57]. Decreased age-related Ca^2+^ buffering capacity and deregulation of Ca^2+^ homeostasis can potentiate excitotoxicity, a phenomenon intimately associated with neurodegeneration [30], [57], [58]. Positioning and ability of mitochondria to move is important for proper Ca^2+^ buffering [59], [60]. We have found that embryonic neurons from all FAD mice exhibited a higher level of cell death in response to the NMDA treatment. However, neurons from PS1 and APP/PS1 mice were affected to the higher extent suggesting that altered mitochondrial motility predisposes neurons to excitotoxic cell death.

Analysis of the pattern of mitochondrial motion also revealed a mutation-specific impact. Thus, only in PS1 neurons mitochondria covered significantly shorter distances between stops. This is consistent with the reported microtubule destabilization caused by increased phosphorylation of cytoskeletal proteins observed in FAD-linked PS1 mice [21], [61], [62]. However, changes in mitochondrial length and distribution were prominent in all three FAD mouse models and already observed in embryonic neurons. The significant increase in mitochondrial length in APP, PS1 and APP/PS1 mice correlates with the reduced numbers of organelles in neuropils. The dynamic relationship between the number of mitochondria in neuropils and mitochondrial length may represent the attempt to maintain constant or increased mitochondrial mass to ensure adequate energy supply. We have also observed the dramatic change in the shape of mitochondria in the brain of APP and APP/PS1 mice. To our knowledge, this is the first report of such structures acquired by mitochondria in AD animal models. Interestingly, the dramatic changes in mitochondrial shape observed only in APP and APP/PS1 mice can't be explained by altered expression of fission and fusions proteins shown to be implicated in AD before [9], [63], [64]. The mechanism involved in the formation of such structures remains to be determined. Examination of the ultrastructure of synaptic mitochondria revealed the increased presence of organelles with altered morphology in all FAD mice starting at 8 weeks of age.

Not surprisingly, alterations in mitochondrial distribution and morphology coincide with the loss of mitochondrial oxidative activity in the brain of all three FAD mouse models, which preceded the onset of amyloid plaque formation and memory phenotype [23], [27], [65], [66]. Taken together, our data suggest that individual FAD mutations facilitate loss of mitochondrial motility, alterations in organelle distribution, loss of morphology and function, which are the early events associated with AD progression.

Application of metabolomics, a global biochemical approach to reveal disease-specific signature of metabolic perturbations, confirmed that mitochondrial function and cellular energy metabolism were altered in all three FAD mouse models. Metabolomic signature of mitochondrial dysfunction was detected in APP/PS1 mice at 16 weeks and in PS1 and APP mice at 8 months of age prior to the formation of amyloid deposits or the onset of memory deficit. Data analysis using PLS-DA showed distinct metabolomic phenotypes in brains of PS1, APP and APP/PS1 mice. Metabolomic signatures of 15 most important metabolites in group separation included molecules linked to mitochondria and energy metabolism, such as inorganic phosphates (Pi), creatinine, NAA, AMP, adenosine, malonic acid, IMP, adenine, β-alanine, lactic acid, ATP, and glycerol. Metabolic pathway analysis revealed that in all three FAD mouse models there are significant alterations in the levels of metabolites involved in energy metabolism including nucleotide metabolism, mitochondrial Krebs cycle, carbohydrate, and amino acid metabolic pathways, which is in agreement with studies conducted in patients and AD mice [67]–[70]. However, along with the pathways equally affected in all three FAD mouse models, we identified metabolic pathways and metabolites that were affected differentially. Thus, alteration in lipid metabolism and energy transfer pathway was specifically observed in APP and PS1 mice, while alterations in glycolytic pathway were prevalent in APP/PS1 mice. Also, mitochondrial Krebs cycle and amino acid metabolism were affected to a greater extent in APP/PS1 mice compared to PS1 and APP mice indicating synergistic effects of both mutations. We have also found that APP/PS1 mice display marked gender differences with female mice having greater metabolic disturbances similar to that observed in human AD patients [71].

Discrimination analysis of individualized metabolomic profiles between single and double mutant FAD mice in simultaneous comparison identified panels of metabolic biomarkers that have predictive power for distinction among three groups, i.e. NTG, PS1 and APP/PS1 and between NTG, APP and APP/PS1 with different degree of AD progression. Metabolites that were uniquely affected in APP mice included panthotenic acid while threonate and ethanolamine were uniquely affected in APP/PS1 mice. Thus, metabolomic signatures of PS1, APP, and APP/PS1 mice indicate individual differences and common metabolic traits in disease development and progression. In all FAD mice, levels of NAA, allo-Inositol and creatinine, molecules that are currently used for the diagnosis of mild cognitive impairment and AD in humans using ^1^H MRI [37], were also affected indicating similarity with human AD. Taken together, our data demonstrate the presence of distinctive changes in mitochondrial motility, dynamics and morphology that correlate with the changes in the levels of metabolites reflecting altered energy metabolism and mitochondrial dysfunction in brain of FAD mice. These changes are mutation specific and could be used for an early diagnosis and monitoring the disease progression. Thus, AD could be viewed as mitochondrial movement disorder with evolving energetic deficit epitomized in the panel of metabolomic biomarkers.

## Materials and Methods

### Animals

Three transgenic mouse lines carrying mutations associated with FAD were used in the study. The APP mice were heterozygous transgenic mice (C57B6/SJL, I.D. No. Tg2576) that expressed mutant human APP695 containing a double mutation (K670N, M671L) [23]. The PS1 mice were homozygous transgenic mice (Swiss Webster/B6D2; I.D. No. M146L) that express mutant human PS1 containing a single mutation (M146L) [26]. The double transgenic mice, APP/PS1, were produced in house by crossbreeding of homozygous PS1 and heterozygous APP mice [28]. The animals were genotyped for the expression of both transgenes by a PCR method using a sample of mouse tail DNA. Littermates that did not carry transgenes were used as non-transgenic control (NTG). All procedures were performed using humane and ethical protocols approved by the Mayo Clinical Institutional Animal Care and Use Committee, in accordance with the National Institute of Health's *Guide for the Care and Use of Laboratory Animals*.

### Preparation of neuronal cultures

Preparation and culturing of primary hippocampal (Hip) and cortical (Ctx) neurons were performed as described previously [72]. Briefly, mice were anesthetized with isoflurane on gestational day 17–18 and fetuses were rapidly removed. Fetal brains were extracted and placed in sterile HEPES- buffered saline (HBS) (pH 7.3). The hippocampi were dissected from each embryo and treated separately. Tissue from each embryo was collected for genotype identification using PCR. Hippocampi were placed in 1 mg/mL papain (Warthington, NJ) in HBS for 20 min at 37°C. After two washes in HBS, the dissociated tissue was triturated in Dulbecco's modified Eagle's medium (DMEM) containing 10% Ham's F12 with glutamine (Gibco/BRL, Grand Island, NY), 10% heat inactivated fetal calf serum (Hyclone Laboratories Logan, UT) and 1× pen/strep antibiotic mixture. Cells were counted, diluted to 3×10^5^ cells/mL, and 2 ml of this stock was placed in each well of a 6-well dish containing glass coverslips coated with poly-L-ornithine (1 mg/2 mL sterile borate buffer, pH 8.4). Plated cells were maintained in an incubator with 5% CO_2_ at 37°C. After 72 h in culture, serum-containing medium was replaced with a serum-free Neurobasal-based medium (without glutamine, Gibco/BRL, Grand Island, NY) containing 1×pen/strep antibiotic mixture and 1×B27 supplement (Gibco/BRL, Grand Island, NY). Quantification of neurons and glial cells using specific antibody staining (GFAP for astrocytes, AB5804, Millipore, and neuron specific βIII-tubulin, ab18207, Abcam) demonstrates that neurons represent 95% of cells present on the coverslip. In cases where experiments required especially pure neuronal cultures, cells were treated with cytosine β-D-arabinofuranoside (Ara-C, Sigma, MO) to a final concentration of 2 µM after 3 and 5 days in culture to suppress proliferation of the glial cells. Such conditions resulted in obtaining fully developed pure Hip neurons exhibiting synaptic activity as judged by staining with synapsin antibody (ab8, Abcam).

### Real time imaging of axonal trafficking in live neurons

Experiments with mitochondrial trafficking were performed as described previously [29]. Briefly, neurons after 7 days in culture (DIC) were treated for 15 min with TMRM (Molecular Probes, Eugene, OR) (final concentration 50 nM). TMRM was washed away with fresh F-12K medium prior to imaging. The experiments were performed using confocal microscope LSM 510 (Carl Zeiss Inc, Germany) with a Plan-Apochromat 100× (1.4 na) oil objective. Cells were incubated at 37°C during the time of recording. All recordings were started five minutes after the coverslip was placed on the microscopic stage to allow equilibration of the sample. Laser was set up to 543 nm for excitation; emission was collected at 585 nm and greater. Axons were selected based on the lack of branching through the whole length. In some cases, verification of neurite identity was made by applying specific antibodies to distinguish dendrites from the axons. A total of 600 images were recorded per cell. Images were taken every 1 sec at highest scan speed (0.9 sec) for 10 min. Three different cells were imaged from one coverslip. Movies were analyzed using LSM 510 software that allowed animation of 600 images into a “movie”. Along with the channel that recorded fluorescence images of moving mitochondria, we recorded axonal movement of the vesicles that were clearly visible in the bright field and were not stained with TMRM. For analysis of axonal trafficking of mitochondria and other vesicles, each organelle was traced from the first frame of the movie to the last. We recorded time and distance that particular mitochondrion traveled in axons, and calculated velocities in anterograde and retrograde directions. We also analyzed the pattern of motion for each mitochondrion. Thus, we estimated whether Mito moved in smooth or stop-and–go mode, what the distances were that Mito covered between stops, and how often did it stop and change direction. We also estimated the fraction of time each Mito spent in motion or stationary state during the time of observation. Data were analyzed for each genotype. At least 22 neurons from 3–5 independent platings were analyzed for each genotype. In some cases, axonal trafficking was analyzed using analytical software Analyze where each Mito was traced through all 600 frames producing kymographs (Figure 1D).

### NMDA treatment

Primary Hip or Ctx neurons (E17) from WT mice were plated as described above. Six days after plating, neurons were switched to DMEM medium without Ca^2+^ and Mg^2+^ ions. Cells were treated with different doses of NMDA (1–80 µM) for 5 min. Cells were washed 3× with (DMEM without Ca^2+^, Mg^2+^) media and returned to the incubator for 24 hrs. Cells were scraped and spun down; medium was aspirated; and cells were re-suspended in 100 µl of fresh DMEM medium. Cell viability was examined using trypan blue staining. Experiments were repeated four times in triplicates with different neuronal platings.

### Immunohistochemistry

The brains from one-day old neonatal APP, PS1, APP/PS1 and NTG mice were immersion fixed with neutral-buffered 10% formalin. Twenty-five micron sections were cut on a cryostat and thaw-mounted on gelatin coated slides. Three sets of adjacent sections from each mouse underwent immunohistochemistry with two different anti-fibrillar Aβ mouse monoclonal antibodies (4G8, 1∶1000, SIG-39220; 6E10, 1∶1000, SIG-39320; Covance Research Products, Berkeley, CA) and an anti-oligomeric Aβ rabbit polyclonal antibody (A11, 1∶5000, AB9234; Chemicon/Millipore, Temecula, CA) using standard ABC immunoperoxidase methods (mouse monoclonal - Vector M.O.M. Peroxidase Kit, PK-2200; rabbit polyclonal – Vectastain Elite ABC, PK-6101; Vector Laboratories, Burlingame, CA). The sections were dried overnight in an oven at 37°C. The sections were then rehydrated with 0.3% Triton X in phosphate-buffered saline (PBST). Endogenous peroxidase activity in the sections was quenched by reacting with 0.5% H_2_O_2_ in PBST for 30 min. The sections were blocked with M.O.M. blocking solution (mouse monoclonal) or 10% goat serum (rabbit polyclonal) in PBST for 60 min. The sections were incubated with the primary antibody at the designated dilution in 0.1% BSA/0.3% Triton-X/PBS overnight at 4°C. The second day, sections were rinsed twice with PBST for 5 min each and then incubated with the appropriate biotinylated secondary antibody at a dilution of 1∶250 for 60 min. After rinsing twice in PBST for 5 min each, sections were incubated with the ABC reagent for 60 min and then rinsed twice in PBS (minus 0.3% Triton X-100) for 5 min each. Sections were reacted with peroxidase substrate (VIP; SK-4600; Vector Laboratories) for an equal amount of time and were then rinsed three times in tap water for 5 min each. Sections were then dehydrated with successive changes of ethanol and xylene and coverslipped.

### Densitometry measurements

Using grayscale images with black equal to 0 and white equal to 255, the mean gray level intensity was measured in three 100 µm×100 µm reticles in cortex and CA1 hippocampal subfield of neonatal NTG, PS1, APP, and APP/PS1 mouse brain. All histochemical conditions, as well as microscope and camera settings were kept constant between all brain sections. The intensity values were normalized against the background intensity of the bare glass slide adjacent to the tissue sections. The normalized values are expressed as ratios with lower values indicating darker, more intense antibody staining and therefore higher levels of Aβ. The normalized intensity ratios for 4G8 immunostaining of Aβ for the cortex and CA1 of the NTG mouse brain were 0.95±0.01 (mean ± SEM) and 0.94±0.01, respectively. The normalized intensity ratios for the cortex and CA1 of the PS1 mouse brain were 0.93±0.01 and 0.93±0.00, respectively. The normalized intensity ratios for the cortex and CA1 of the APP mouse brain were 0.92±0.00 and 0.89±0.00, respectively. The normalized intensity ratios for the cortex and CA1 of the APP-PS1 mouse brain were 0.86±0.01 and 0.75±0.01, respectively. The normalized intensity ratios for A11 immunostaining of oligomeric Aβ for the cortex and CA1 of the NTG mouse brain were 0.93±0.01 (mean ± SEM) and 0.93±0.01, respectively. The normalized intensity ratios for the cortex and CA1 of the PS1 mouse brain were 0.77±0.00 and 0.81±0.00, respectively. The normalized intensity ratios for the cortex and CA1 of the APP mouse brain were 0.80±0.01 and 0.81±0.00, respectively. The normalized intensity ratios for the cortex and CA1 of the APP-PS1 mouse brain were 0.70±0.00 and 0.68±0.00, respectively.

### Western blot analysis

Expression of mitochondrial fission and fusion proteins was detected using the following antibodies: rabbit polyclonal Opa1 (1∶1000, Novus Biologicals), mouse monoclonal Opa1 (1∶1000, BD Transduction Laboratories), mouse monoclonal Dlp1 (1∶1000, BD Transduction Laboratories), chicken Mfn1 (1∶1000, Novus Biologicals), rabbit polyclonal Mfn2 (1∶1000, Sigma-Aldrich), mouse monoclonal Mfn2 (1∶1000, Abnova), rabbit polyclonal Fis1 (1∶500, Alexis Biochemicals), and mouse monoclonal Tim23 (1∶1000, BD transduction Laboratories). Tissue extracts were prepared from either the whole brain or from dissected cerebellum, cortex and hippocampus from APP/PS1, PS1, APP and NTG mice 11–12 months old. Tissue was homogenized and lysed using 1× RIPA Buffer plus inhibitors. 20 and 40 µg of protein from the same sample was loaded in every well. Proteins were separated by SDS-PAGE using 4–20% Criterion Tris-HCl gels (Bio-Rad). Proteins were visualized by fluorophores conjugated with secondary antibodies (ZyMaxTM Goat Anti-Rabbit IgG (H+L) CyTM 5 conjugate and/or Alexa Fluor 488 goat anti-mouse IgG (H+L), Invitrogen) and analyzed using a PharosFX Plus Molecular Imager (Bio-Rad).

### Estimation of mitochondrial mass and activity

NTG, APP, APP/PS1 and PS1 mice 7 months old were sacrificed by decapitation, brains were quickly removed and placed in ice-cold Krebs-Ringer Bicarbonate Buffer (KRB) pH = 7.3 [73]. Brains were embedded in 2.5% agar and cut coronally by Vibratome into 50 µm-thick slices. Each brain slice was incubated for 15 min at RT in either KRB alone or KRB containing MitoTracker Green (MTG) and MitoTracker Orange (MTO) (both from Molecular Probes) as described in [34]. Tissue slices were washed in KRB and imaged using LSM 510 confocal microscope (40× lens; MTG, ex 490/em 516 nm; MTO, ex 551/em 576 nm). Estimation of MTG and MTO fluorescence intensities was done using LSM Physiology software. The ratios between MTG and MTO fluorescence were taken as a measure of mitochondrial activity. At least five images for each genotype were taken into analysis.

### Electron microscopy

For conventional electron microscopy, animals were perfused with 4% paraformaldehyde, brains were removed and post-fixed in Trump's solution overnight. Next day, Hip (CA1 region) was dissected from each brain and subjected to EM staining. CA1 Hip tissue was incubated in 1% osmium tetroxide, dehydrated in a graded series of ethanol and embedded in Quetol 651 (Ted Pella, Inc). Thin sections (0.09–0.1 µm) were cut parallel to the ventral surface using a diamond knife (Diatome US) and an Ultracut E microtome (Reichert-Jung, Wien, Austria). Sections were collected on copper grids, post-stained with lead citrate and viewed at ∼80 kV with a JEOL 1400 transmission electron microscope (JEOL USA). Three to five randomly selected micrographs per each age and genotype were analyzed by blinded investigator. The following parameters were estimated: the number of mitochondria with abnormal shape, the number of mitochondria per neuropil, neuropil length, number of mitochondria per neuropil length, and the average length of mitochondria. The following mice were used for the EM examination: APP/PS1, PS1, APP and NTG of 8, 12, 24, 30, 33, 40, 45 and 64 weeks of age.

### Sample preparation and metabolomic analysis

Metabolic signatures were generated in three female NTG, PS1 and APP mice 36 weeks old, and in the groups of three female and three male NTG and APP/PS1 mice 16 weeks old. Adult mice were sacrificed; brains were rapidly removed; Hip tissue was dissected and flash-frozen in liquid nitrogen. Tissue was pulverized under liquid N_2_ and extracted in a solution containing 0.6 M HClO_4_ and 1 mM EDTA [74], [75]. Extracts were neutralized with 2M KHCO_3_ and used for metabolomic analysis. For gas-chromatograph/mass-spectrometer (GC-MS) analysis, 100 µL of extract was transferred into Eppendorf tube and spiked with 5 µL internal standard (IS), myristic-d_27_ acid, (1 mg/mL) at ambient temperature. After gently vortexing, samples were completely dried in a SpeedVac concentrator. The samples were subsequently methoximated using 20 µL of a 20 mg/mL solution of methoxyamine hydrochloride in pyridine at 30°C for 90 min and then derivatized using 80 µL of N-methyl- N-trimethylsilyltrifluoroacetamide with 1% trimethylchlorosilane (MSTFA+1% TMCS, Pierce) at 37°C for 30 min [76]. Metabolite levels were determined using GC-MS (Hewlett-Packard, HP 5980B) with DB5-MS column and HPLC (Hewlett-Packard, series 1100) with Mono QTM column (GE Healthcare Bio-Sciences AB) using a triethylamine bicarbonate elution buffer (pH 8.8), and a reverse phase C-18 column using a phosphate buffer, tetrabutylammonium sulfate and methanol mixture [77]–[79]. GC-MS spectra were deconvoluted using AMDIS software, after that SpectConnect software was used to create metabolite peaks matrix [80], [81]. The Agilent Fiehn GC/MS Metabolomics RTL Library was used for metabolite identifications. The matrix data were exported to SIMCA-P+ software (v12.0, Umetrics, Umea, Sweden) for multivariate data analysis. Unsupervised principal component analysis was run to detect any innate trends and potential outliers within the data. Supervised partial least squares discriminant analysis (PLS-DA) was performed to obtain additional information on differences in the metabolite composition of groups. PLS-DA models were calculated with unit variance scaling, and the results were visualized in the form of score plots to show the group clusters. The VIP (variable importance in the projection) values and regression coefficients were calculated to identify the most important molecular variables for the clustering of specific groups. The PLS-DA model was validated by comparison to the classification statistics of models generated after random permutations of the class matrix [82], [83].

### Statistical Analysis

Statistical analyses of means for more than two groups were performed using one-way analysis of variance (ANOVA) with the categories of genotype and age as independent factors followed by the Newman-Keuls post-hoc test for multiple comparison. For analyses of means involving only two groups with a sample size n<30, the F-test was used to determine if the variances between the two groups were significantly different. For samples with a significant difference in variance, the Welch's *t* test was applied. Student's *t* test was applied for the samples with an insignificant difference in variance of where n≥30. The null hypothesis was rejected at the 0.05 level. All statistical computations were carried out using Prism (Graphpad Software). Results for the vesicular motility and length are expressed as the mean ± S.D. For multivariate data analysis, unsupervised principal component analysis (PCA) and supervised PLS-DA were done using SIMCA-P+ software (v12.0, Umetrics, Umea, Sweden). The details of data analysis implemented in metabolomic study are described above.

## Supporting Information

Movie S1 **Axonal trafficking of mitochondria in primary neuron from NTG mouse.** Visualization of mitochondria in E17 Hip neuron was done using TMRM. 600 frames were acquired by imaging the axon every second using LSM 510 confocal microscope. Imaging was done focusing on the axon with the cell body located at the top of the image. Resulting movie was analyzed using Analyze, a comprehensive multidimensional medical image processing, visualization and analysis software package developed by the Biomedical Imaging Resource of the Mayo Clinic [84]. By treating the microscope image sequence as a spatial stack of cross-sectional images, the volume rendering algorithms in Analyze produce a 3D digital kymograph, allowing the motion of multiple organelles over a period of time to be visualized in a single static 2D image. Final kympgraph allows tracing each mitochondrion through all 600 frames to generate a final profile of movement.
(MPG)

Movie S2 **Animated 3D reconstruction of mitochondrial structure in Hip tissue of APP/PS1 mouse 24 weeks of age.** For 3D reconstruction of the mitochondrial structure, the grayscale of the individual EM section images was first inverted so that the organelle became bright objects. The inverted images were then sequentially co registered using the Normalized Mutual Information 2D registration program in Analyze [84]. This is an automated procedure that aligns similar images based on the statistical distribution of paired pixels compared to the distribution in either image alone. The inverted, co-registered stack was then rendered using Maximum Intensity Projection. Each pixel in the rendered image represents the brightest voxel in a ray from the viewers' eye through the entire stack of sections.
(MPG)
